# Quantifying Market Trajectories: A Study of Pharmaceutical Sales Uptake Curves and Related Parameters

**DOI:** 10.1007/s43441-026-00954-8

**Published:** 2026-06-17

**Authors:** Irwin Tendler, Shomesh Chaudhuri, Chinmay Shukla, Neil Kumar, Andrew Lo

**Affiliations:** 1 BridgeBio (United States), Palo Alto, USA; 2 QLS Advisors, Cambridge, MA USA; 3 Sloan School of Management, Massachusetts Institute of Technology, Cambridge, USA

## Abstract

This study provides a comprehensive view of the dynamics of pharmaceutical sales trajectories within the United States market, drawing from extensive historical sales data covering 391 product launches from January 1, 2000 to December 31, 2023. We analyze key performance metrics including, but not limited to, peak annual sales volumes, time-to-peak measurements, and annual growth rates. In addition, we provide detailed segmentation analysis across multiple dimensions, including therapeutic areas, treatment modalities, company profiles, and regulatory designations. To our knowledge, in comparison to the published literature, our analysis constitutes the most systematically stratified survey of US pharmaceutical sales trajectories. Our analysis demonstrates significant variability in sales uptake patterns and highlights factors such as therapeutic context, modality type, sponsor characteristics, and regulatory pathways that influence these dynamics. The findings provide novel quantitative benchmarks and practical insights for biopharmaceutical forecasting and strategic decision-making.

## Introduction

Understanding net sales trajectories for pharmaceutical products is a critical component of biopharmaceutical business strategy and decision-making. These trajectories, which map out the revenue potential of drugs from launch through patent expiry, serve as fundamental indicators for multiple stakeholders including investors, executives, and research teams.

The analysis of uptake curves, including parameters such as peak sales—the highest annual revenue a drug achieves during its lifecycle—remains challenging as it has not been analyzed comprehensively across the Pharma industry. While exceptional cases like Lipitor’s peak annual worldwide sales of ~ $14B and Humira’s ~ $22B are often referenced, these represent outliers rather than typical performance metrics (median PYS are expected to be ~ $500–$800 M [1–3]). Although peak sales projections are critical in shaping how companies allocate their resources and prioritize their pipelines, the industry lacks standardized and accurate metrics for evaluating the more representative sales outcomes. [4–6] Moreover, peak sales are frequently used by investors to assess the value of pharmaceutical companies, thereby influencing investment decisions [7–9]

Beyond peak sales considerations, sales curve dynamics encompass four critical phases: the launch period (years 1–3), growth phase (years 3–5), maturity (years 5–10), and eventual decline, which typically coincides with patent expiration and generic entry [10]. Understanding these patterns enables companies to make strategic investment decisions throughout the product lifecycle, from initial research through manufacturing and sales force deployment. Furthermore, companies rely heavily on commercial potential when prioritizing R&D efforts, selecting therapeutic areas and development candidates based on projected sales figures and market opportunities. These sales estimates also guide crucial lifecycle.

management strategies, including decisions about patent extensions, new formulations, and geographic expansion plans. A survey of the literature shows that the mismatch between sales predictions, versus eventual actual reported values, often differ by > 50–70% [11]

In recent years, modern analytics and integrated datasets have enhanced our ability to predict and understand pharmaceutical market trajectories, making them essential components of business planning and strategic decision-making. While previous research has extensively examined technical success probabilities, particularly in clinical development [12–18], there remains a significant gap in the systematic analysis of sales uptake patterns across different therapies and modalities. Despite the existence of individual case studies for prominent drugs like Humira and therapeutic classes such as checkpoint inhibitors, comprehensive analyses of launch trajectories and market penetration dynamics are notably scarce [19, 20].

To address this knowledge gap, our analysis leverages comprehensive pharmaceutical industry data by integrating two sources: Evaluate Pharma and Biomedtracker. Through this combined dataset, we examine 391 NME drug launches (inclusive of biologics approved via CBER BLA filing) spanning over the course of the last 25 years. By analyzing historical net sales data, we characterize key performance metrics including peak sales, time to peak, growth trajectories, and other critical curve attributes across these historical launches. We conduct detailed stratification using multiple filters, encompassing disease area, modality, sponsor type, and regulatory pathway. This approach yields quantitative benchmarks and identifies factors that shape commercial sales curves across diverse market segments and product categories – these benchmarks can be directly used to inform forecasting models or strategic planning.

## Data and Methods

Our analysis primarily sources historic company financial data from Biomedtracker (BMT, Norstella Companies, Yardley, PA). We also obtain essential drug metadata – including indication, modality, approval year, sponsor, and regulatory pathway – from BMT’s comprehensive pharmaceutical database, which provides standardized and aggregated drug development information. For instances of data missingness, we improve the completeness of our database by integrating Evaluate Pharma (Norstella Companies, Yardley, PA). Drug product matching across data sets is completed using exact matches on brand name and supplemented with manual confirmations of highly similar brand name matches as determined by algorithmic approximate string matching.

To note, both Biomedtracker and Evaluate Phara utilize primary sources including, but not limited to, company presentations, SEC filings (10-K and 10-Q reports), annual reports, and other public financial disclosures to generate a repository of historic sales data for both US and outside-US geographies. We adjusted all sales figures in our database to be inflation-adjusted on an annual basis utilizing the NIH Biomedical Research and Development Price Index [21–23].

The inclusion and exclusion criteria for this analysis have been defined to maximize industry-specific relevancy:

*Inclusion criteria*: therapeutic drug products with a minimum of 5 contiguous years of verifiable sales data, providing a substantial track record of market performance in order to enable a robust longitudinal performance analysis.

*Exclusion* criteria: To maintain focus on new drug launches, this analysis excludes medical devices, diagnostics, generics, and biosimilars.

In analyzing sales performance trajectories, we identified a set of key variables which commonly serve as critical indicators of market success. Peak annual sales provide an essential benchmark for understanding a product’s maximum market penetration. Time-to-peak, measured in years from launch to 90% of peak value (accounts for the point in time during which increasing annual price change becomes the larger driver of increasing sales), reveals insights into market adoption speed and product lifecycle patterns. Year-over-year sales growth rates, measured as annualized growth rate between sales in the first full year post-approval (“Year 2”) and sales in Year 5,^1^ can serve as predictors of long-term performance and indicate initial market acceptance. Sales growth acceleration, calculated as the difference of the sales growth between Years 4 and 5 and the sales growth between Years 2 and 3, identifies critical inflection points and evolving market dynamics throughout a product’s commercial lifecycle. For example, if sales growth between Years 2 and 3 is 50% and slows to 10% between Years 4 and 5, the resulting acceleration would be − 40%, indicating a deceleration and potential early signs of market saturation. Together, these interconnected variables form a robust framework for evaluating and characterizing product launch performance.

Descriptive statistics (mean, median, and distribution) of each metric are generated, alongside systematic stratification across disease area, drug modality, sponsor type and regulatory pathway.

Disease areas serve as a primary segmentation method, with classification into therapeutic areas including oncology, immunology, central nervous system disorders, and infectious diseases. Treatment modalities form a second stratification layer, encompassing modalities such as small molecules, antibodies, and gene therapies. Sponsor type segmentation distinguishes between established pharmaceutical corporations and emerging biotech companies. The final analytical dimension focuses on regulatory features, examining key designations such as accelerated approval and orphan status. This systematic, multi-layered approach enables thorough examination of interconnected patterns across distinct dimensions.

To identify distinct patterns in product sales trajectories, we employed K-means clustering on normalized sales data for the first five years post-launch. For normalization, we divided the first five years’ sales by the product’s peak sales value. This normalization approach preserved the relative shape of each sales curve while standardizing their scale. We applied K-means clustering (K = 4) to the normalized 5-year sales profiles, resulting in four distinct sales trajectory archetypes: (1) “Early, Sharp Peak,” characterized by rapid initial uptake followed by decline; (2) “Fast Uptake, Early Plateau,” showing quick adoption that plateaus early; (3) “Moderate Uptake,” displaying gradual growth to peak within the 5-year period; and (4) "Prolonged Uptake," exhibiting continuous growth throughout the five-year period and beyond. We selected K = 4 on the basis of an elbow analysis of within-cluster variance and domain interpretability. To note, increasing to K = 5 did not reveal a qualitatively new uptake archetype but instead subdivided existing clusters along the fast-to-prolonged uptake continuum, while K = 3 merged commercially distinct patterns.

## Results

### Overall Characteristics of Sales Curves

#### Peak Year Sales

**Fig. 1:**
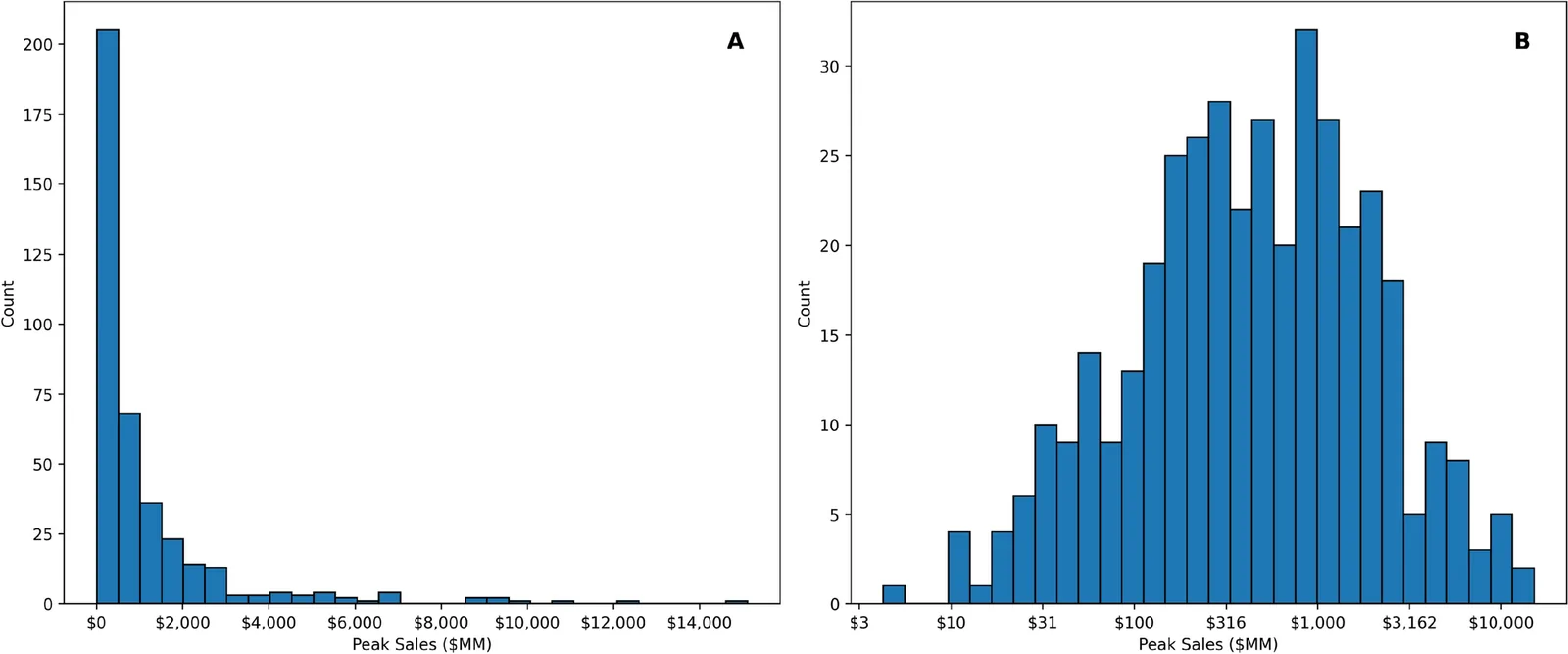
A) Histogram of US peak sales for all drug products in our database meeting inclusion criteria. For n = 391 entries; μ = $1132 M ± $1870 M; min/5th/25th/median/max75^th^/95th/max = $3 M / $30 M / $159 M / $462 M / $1,244 M / $4,833 / $15,114 M B) Same data set shown under a log(10) transformation

Our aggregate analysis examines patterns across all drug launches selected via our inclusion criteria (n = 391). We begin by examining US peak sales outcomes. Figure 1 presents a histogram illustrating the distribution of maximum sales achieved across products. The analysis reveals a mean of $1132 M and a median of $462 M, with the distribution showing significant positive skewness.^2^

To better visualize the wide range of outcomes, particularly given the substantial right tail created by several Blockbuster drugs, Fig. 1B presents the same data using a logarithmic transformation. Effectively, this figure shows a compression of extreme right-tail values and reveals a more normalized distribution spread over multiple orders of magnitude. We note that the data in Fig. 1B visually represents a normalized distribution pattern.

#### Time-to-Peak Year Sales

**Fig. 2:**
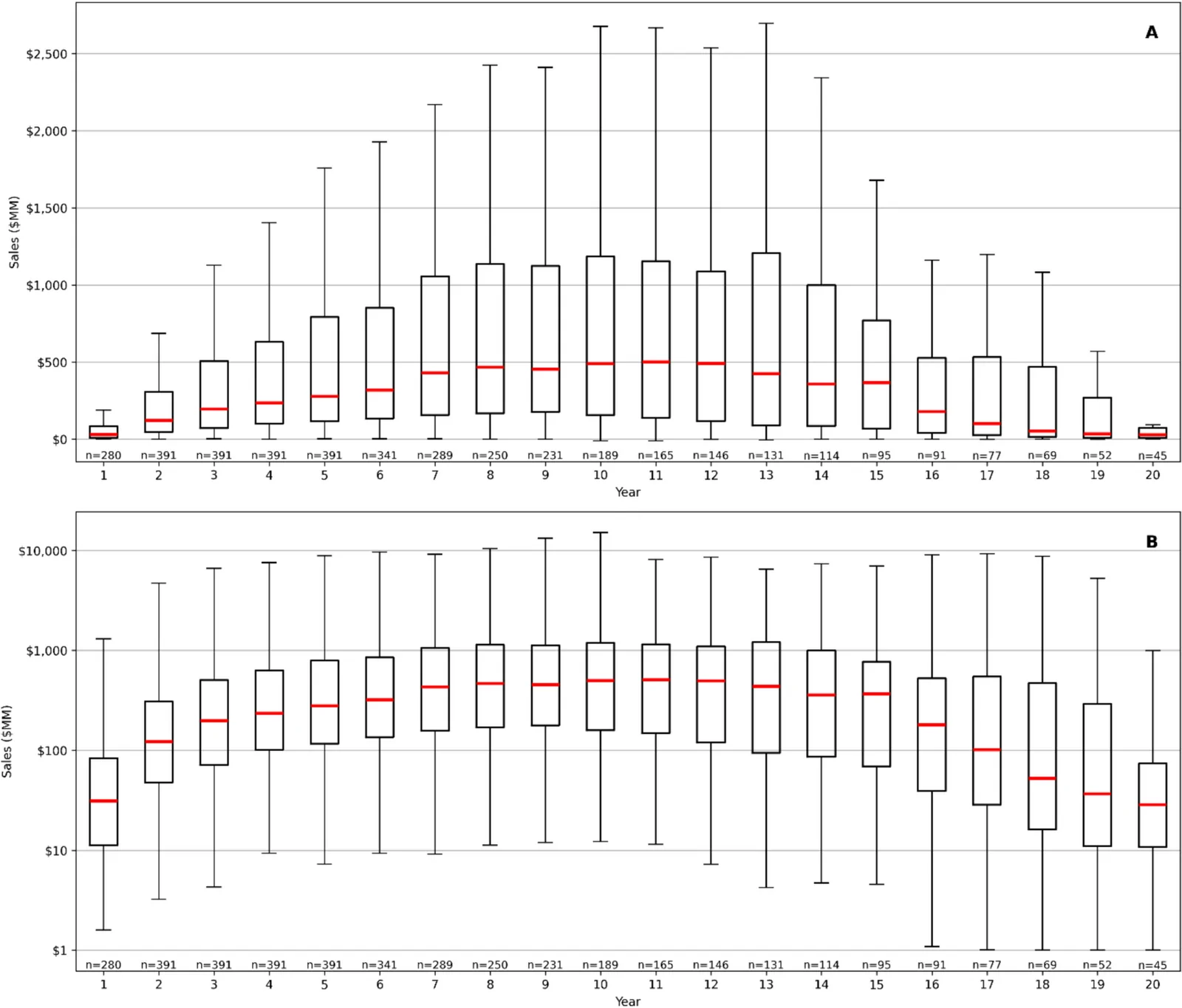
**A** Box plots of inflation-adjusted US peak sale distributions for all drug products in our database meeting inclusion criteria, per year from launch**.** Lower black-colored horizontal bar is minimum value, 25th percentile is lower range of yellow box, red horizontal bar is median, 75th percentile is upper range of yellow box, upper, black-colored, horizontal bar is maximum value.** B** Same data set shown as distribution of log10(1 + sales) over 20 years

Next, we explored the time-based profiles of annual sales. Figure 2a illustrates the spread of annual worldwide sales by year from launch using box-and-whisker plots for all products meeting our inclusion criteria. Median time to peak year sales is achieved at ~ 5 years from the first full launch year. Once peak sales are attained, we found that sales figures plateau at the same level for an average of ~ 7 years thereafter (before appreciable decline begins).

The sales distributions exhibit marked asymmetry in their non-log-scaled form (Panel A), displaying a pronounced right skew with extended upper tails that indicate exceptional performers significantly outpacing median sales. Upon log transformation (Panel B), these distributions become notably more symmetric, consistent with the log-normal distribution in peak sales observed in Fig. 1.

**Fig. 3:**
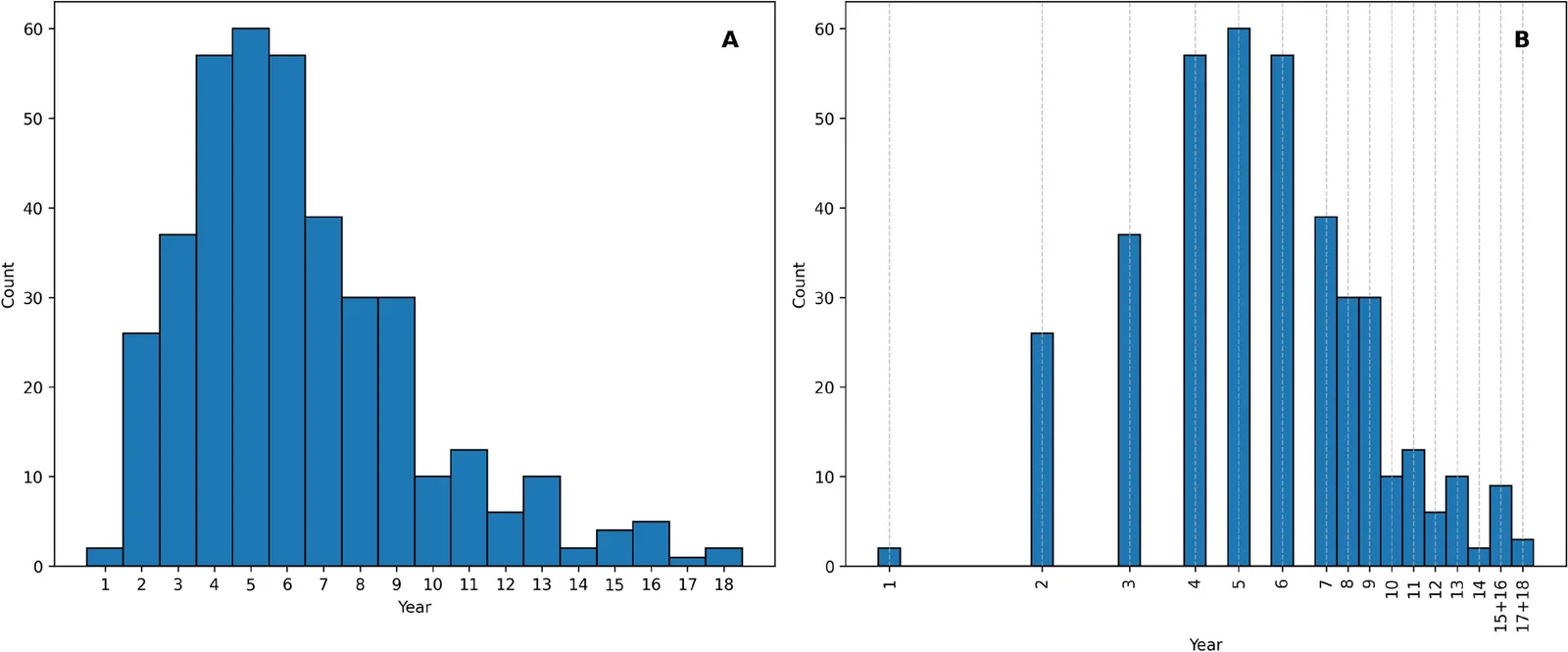
Histogram of time-to-peak for all drug products meeting inclusion criteria in both normal (**A**) and log(10) transformed (**B**) spaces. Almost half (189 of 391; 48.3%) reach peak within 5 years, but a tail of slow burners take at least 10 years (53 of 391; 13.6%) to hit peak sales. For n = 391 entries; μ = 6.35 ± 3.21 years; min/5th/25th/median/max75^th^/95th/max = 1 year / 2 years / 4 years / 6 years / 8 years / 13 years / 18 years

In Fig. 3, we find that almost half (182 of 391; 46.5%) drugs reach peak year sales within 5 years post-launch; however, the distribution of time required to reach peak is skewed to the right with an extended tail again suggesting a log-normal distribution (see Panel B). This implies that for a subset of drug products (53 of 391; 13.6%), at least 10 years are required prior to reaching peak year sales. Overall, we found a mean time-to-peak of 6.4 years, with a standard deviation of 3.2 years. The fastest 5% of products peaked within 2 years, whereas on the other extreme, about 5% took at least 13 years to peak. This range reflects the effects of indication expansion vectors and diverse market dynamics, from rapid adoption in high-demand areas to prolonged uptake in others.

#### Sales Growth Rates & Acceleration

**Fig. 4:**
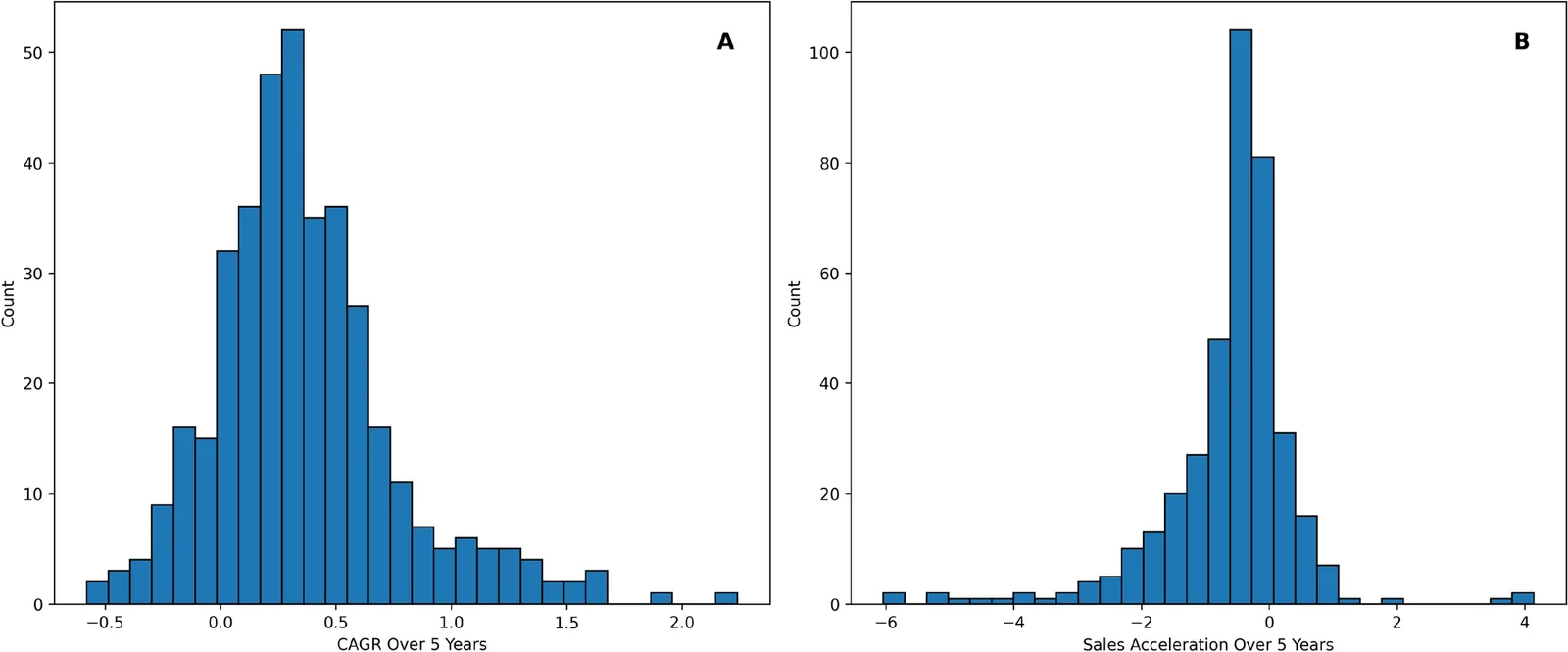
**A** Histogram of product 5–yr CAGRs.** B** Histogram of sales acceleration over 5 years from product launch. Note, for A) and B), outliers below the 1st and 99th percentiles were filtered out resulting in an n = 383/391. For n = 383 entries in (**A**), CAGR μ = 37 ± 41%; min/5th/25th/median/max75^th^/95th/max = − 57% / − 19% / 12% / 54% / 116% / 224%. For n = 383 in (**B**), sales acceleration over 5 years μ = − 0.6 ± 1.1%; min/5th/25th/median/max75^th^/95th/max = − 6% / − 2% / − 1% / − 0.4% / − 0.01% / 0.5% / 4.1%

In addition to peak sales and timing, we examined early growth rates and acceleration of sales, which provide insight into the shape of uptake curves. Focusing on the initial trajectory, we calculated each product’s compound annual growth rate (CAGR) over launch years 2 (1st full year of reported sales) through 5, Fig. 4a. Analysis showed that outliers within the top and bottom 1% percentiles (representing a minor subset of eight product launches, when categorized by 5-year CAGR) heavily skewed data, and as such, were excluded from this sub-analysis. Following exclusion of these extreme outliers, the median annualized growth rate was 31.1% per year. The 25th to 75th percentiles of products (interquartile range) showed CAGRs from 12.0% to 54.3% per year over their first 5 years. To that end, 28.9% (113) of products experienced extremely rapid early growth, exceeding 50% CAGR in that period. On the other end, 14.3% (56) of products had flat or negative growth in the first few years (often products that peaked immediately or faced setbacks). This wide IQR (12.0% to 54.3%) indicates that while the “typical” new drug shows strong double-digit growth post-launch, there is high variability. Notably, a small subset (14.3%) of products exhibited flat or declining sales after the first full year of sales — these are cases where initial sales were high and subsequently dropped. Potential drivers of this phenomenon are intrinsic product characteristics (e.g., one-time gene therapy injection which rapidly penetrates, and thereby exhausts, its addressable population) or serious issues which emerge post-launch and curtail uptake.

Regarding sales acceleration, we interpret a positive acceleration as an increasing growth rate each year (an uptake curve that is concave up), and a negative acceleration as a decreasing growth rate over time (concave down). After removing outliers, the median launch was slightly decelerating (− 42.9%), suggesting that, typically, growth tends to slow down across the first 5 years of sales (see Fig. 4b). However, to note, to the distribution shows a wide range of divergent patterns: 19.7% (77/391) of products had positive acceleration (their growth rate between years 4 and 5 was higher than their growth rate between years 2 and 3), whereas around 23.0% (90/391) showed a deceleration less than − 100% (strong initial ramp that tapered off). The IQR acceleration metric spanned from − 90.5% to − 9.0%, with a mean of − 61.4% and standard deviation of about 108.2%. In practical terms, we see a variation in sales trajectories with some products launching with strong growth that slows over the first 5 years (deceleration), whereas others that start with more modest growth rates managed to accelerate as they gained traction in the market – persistence/durability in positive sales acceleration inevitably leads to, comparably, heightened PYS. The presence of both patterns indicates there are distinct archetypes of uptake curves, which we explore next.

#### Uptake Curve Archetypes

**Fig. 5:**
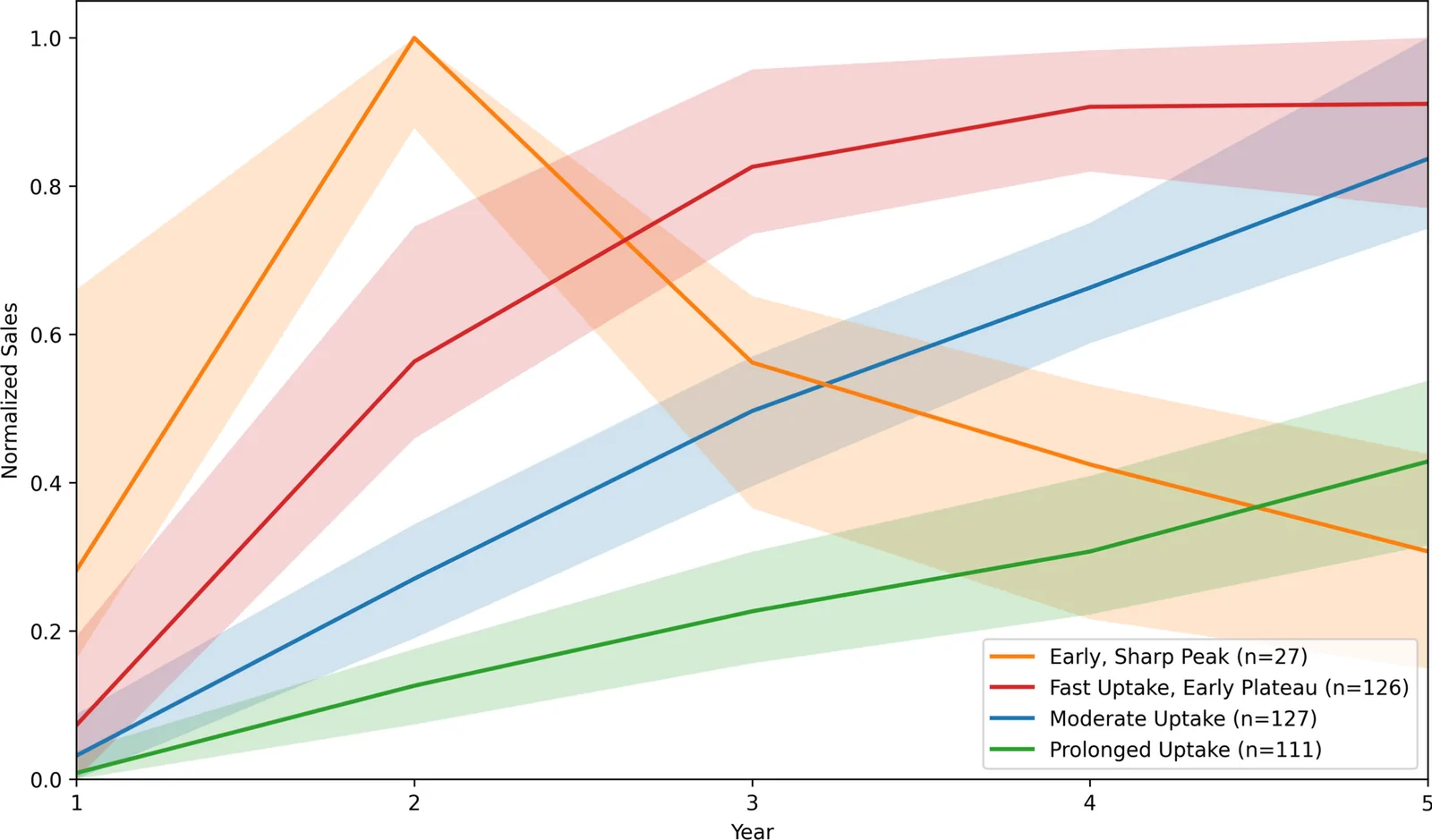
Normalized sales curves for cluster archetypes (annual sales / peak sales; years 1–5 post-launch). Number of drug products per cluster: "Early, Sharp peak” = 27, “Fast, Uptake, Early Plateau” = 126, “Moderate update” = 127, and “Prolonged uptake” = 111. Solid line represents median, light shading for upper/lower bounds are 25th–75th percentiles

We applied an unsupervised clustering algorithm (k-means) to the time-series sales data, shown above, to identify common patterns/archetypes of sales trajectories. This analysis yielded four distinct clusters of product launches, each representing a characteristic uptake curve shape (see Fig. 5):

1. “Early, Sharp Peak”, a rapid ramp-up to peak sales very early after launch, followed by a noticeable decline (example product, Harvoni). About 7% of products (n = 27) fell into this cluster. These are cases where drug sales tended to peak within the first two years of launch and then decreased, possibly due to early saturation of the market or the entrance of competition or safety issues. Such products often had an initial surge (e.g., due to unmet need), but could not sustain growth. The “Early, Sharp Peak” cluster had a median time to peak of 2 years and, interestingly, a relatively high average peak sales ($1.54B), suggesting that when this pattern occurs, it could often involve a large initial bolus of patients whose needs are quickly fulfilled before transitioning to treating incident patients. Note, at the 2 years post-launch, the upper-quartile range becomes quite narrow, which is both a function of our smaller sample size (n = 27) and intrinsic commercial success for this archetype
2. “Prolonged Uptake”, a gradual increase in sales over an extended period, often reaching a high peak late in the product’s lifecycle (example product, WelChol). Approximately 28% of products (n = 111) showed this pattern. These drugs started with modest sales, but kept growing year after year, sometimes aided by new indications, expanded patient access, or growing clinical acceptance. Their median time-to-peak was 9 years—the longest of any cluster—and they achieved the highest mean peak sales ($1.79B) among clusters. This indicates that extended sales momentum can coincide with strong market performance. Often, products in this cluster are therapies which gradually expand their use cases, or those that steadily penetrate a broad population
3. “Moderate Uptake”, a “middle-of-the-road" trajectory characterized by moderate early growth and a peak at 6 years (median). This was the largest cluster, including about 32% of products (n = 127). These sales curves increase steadily and then plateau before declining (example product, Trileptal). The mean peak sales in this group were $914M. Many mainstream small molecule drugs for chronic conditions fall in this category: they show reliable growth and achieve peak sales within 5–7 years, without extreme early surges or extremely prolonged ramp-up.
4. “Fast Uptake, Early Plateau”, a pattern of aggressive early adoption and growth, reaching peak relatively quickly (median of 4 years), but often with more limited peak magnitude (example product, Viread). About 32% of products (n = 126) fit this cluster. These drugs had high initial growth rates (often fueled by strong launch marketing and high unmet need), thereby attaining peak sales within 3–4 years, then typically exhibiting slowing/steady growth and a sustained peak. The mean peak sales for this archetype ($681M) were the lowest of the four clusters. This pattern might describe, for example, a highly effective niche drug that rapidly reaches all patients in its niche. Once the eligible patient pool is on therapy, growth flattens, capping the peak lower than in some other clusters

Each of these archetypes was well-represented in the dataset (detailed statistical analysis shown in Table 1), indicating that there is no single “standard” sales trajectory. Instead, new products can follow multiple paths to market maturity. These patterns help contextualize the earlier aggregate findings: for instance, the presence of the prolonged uptake cluster explains the long-tail in time-to-peak (some drugs in that cluster took a decade or more to peak), whereas the fast uptake and early peak clusters explain why many drugs peak early. Importantly, these results provide a quantitative basis for scenario-based forecasting. If certain product or market characteristics suggest a new launch will follow a given archetype (e.g., an “early sharp peak” pattern for a first-in-class oncology drug addressing high unmet need), one can use the historical distribution of that cluster as a benchmark for expected time to peak and peak sales level.

**Table 1:** Statistical analysis for drug product archetype “clusters”

Key metric	Statistic	Early, Sharp peak	Fast uptake, Early plateau	Moderate uptake	Prolonged uptake
Count		27	126	127	111
Peak sales ($MM)	Mean	$1544	$681	$914	$1794
	Std	$3083	$1293	$1193	$2420
Pecentiles	5%	$20	$24	$46	$68
	25%	$61	$112	$159	$482
	50%	$321	$230	$428	$954
	75%	$1065	$683	$1118	$1930
	95%	$8902	$3623	$3042	$6812
Time to peak sales (Years)	Mean	3.8	4.0	6.2	9.8
	Std	3.6	1.3	1.6	3.0
Pecentiles	5%	1.3	2.0	4.0	6.0
	25%	2.0	3.0	5.0	8.0
	50%	2.0	4.0	6.0	9.0
	75%	4.0	5.0	7.0	11.0
	95%	11.0	6.0	9.0	16.0
5-Year CAGR (%)	Mean	− 34%	15%	62%	62%
	Std	21%	19%	84%	55%
Pecentiles	5%	− 71%	− 15%	16%	1%
	25%	− 46%	4%	31%	31%
	50%	− 30%	14%	46%	51%
	75%	− 23%	24%	61%	79%
	95%	− 4%	41%	137%	143%

### Stratified Analyses

We present an analysis decomposing key sales trajectory metrics by various subgroups (stratifications): disease area, drug modality, sponsor type, blockbuster achievement, and regulatory designations. For each stratification, we report peak sales, time-to-peak, sales acceleration (5–yr CAGR) and distribution of archetypes.

### Disease Area

To examine trends across disease areas, we grouped products into their corresponding indication groups (based on primary approved indication), Table 2. Disease-area specific trends for indications with n > 10 drug products within our database:

- *Immunology/Autoimmune* (n = 33) and *Respiratory* (n = 15) drugs tend to have among the highest typical peak sales (median ~ $852M and $911M, respectively). These fields include many biologics for chronic conditions (e.g., rheumatoid arthritis, asthma) that achieved blockbuster status. The median time to peak is about ~ 7 years for immunology and ~ 5 years for respiratory. Early growth in immunology is strong (median ~ 40% 5–yr CAGR), reflecting robust adoption of effective therapies.
- *Oncology* (n = 95) has much lower median peak sales (~ $419M) despite being the largest category in our database. This likely reflects the proliferation of niche oncology drugs targeting specific tumor types, or small patient subsets. Many oncology launches achieve moderate sales rather than mega-blockbusters. Time to peak is ~ 6 years (median). Oncology drugs often enter crowded markets facing competition from incumbents, which may limit their peak. The 5–yr CAGR median is ~ 20%, one of the lower growth rates, potentially because oncology drugs often see rapid uptake in niche populations, but then usage plateaus due to competition or limited eligible patients.
- *Cardiovascular* (n = 24) and *Psychiatry* (n = 17) drugs show relatively high median peaks (~ $790M and ~ $1129M, respectively) and long times to peak (median ~ 9 years for cardiovascular, ~ 8 years for Psychiatry). These are large primary care markets where new therapies (e.g., novel cholesterol drugs or antidepressants) may face slower uptake due to entrenched standards of care, and the burden of clinical evidence required to prove long-term benefits. The growth rates in early years are ~ 50% for cardiovascular (one of the highest median 5–yr CAGRs), likely because once such drugs surpass initial adoption hurdles, they can expand rapidly in large patient populations. Psychiatry also shows a meaningful ~ 30% median 5—yr CAGR. Overall, the corresponding long ramp-ups in cardiovascular and psychiatry indications are driven by conservative adoption curves and the ultimate need for guideline changes or payer acceptance; however, ultimately large patient pools drive eventual high peak sales once adoption eventually accelerates.
- *Metabolic* (n = 19) and *Endocrine* (n = 33) categories (which include diabetes, obesity, and other metabolic disorders) have intermediate median peaks (~ $484M and $646M) and a median ~ 5–6 year time-to-peak. Notably, these categories contain some of the fastest-growing recent therapies (e.g., GLP-1 agonists for diabetes/obesity) but also many modest growth/PYS drugs. The variability in PYS is high (wide IQR), with a median 5–yr CAGR of ~ 25–40% between the two indications.
- *Infectious Disease* (n = 60) drugs have a median peak of ~ $385M and a fairly quick median time to peak of ~ 5 years. Many anti-infectives (e.g., antibiotics) see rapid uptake if they address an acute need (especially antivirals or new vaccines), but can generate relatively limited long-term revenue due to short treatment durations or competition. The median 5–yr CAGR is ~ 17%, the lowest of any major category, which may indicate that many anti-infective products launch and reach near-peak usage very quickly (e.g., a new antibiotic might be widely used immediately in hospital settings, if effective), implying that there is substantial ongoing growth post-launch i.e. immediate capture of market share, followed by plateau or decline in sales growth rate.
- *Neurology* (n = 41) drugs, for diseases such as multiple sclerosis, epilepsy, Alzheimer’s, etc.), have moderate median peaks (~ $484M), supported by ~ 6 years to peak and median 5–yr CAGR of ~ 40%. This suggests that these products are strong launchers with early-moderate level growth

**Table 2:** Statistical analysis for drug product indication stratification

Key metric	Statistic	Allergy	Autoimmune/immunology	Cardiovascular	Dermatology	Endocrine	Gastroenterology (Non IBD)	Hematology	Infectious disease	Metabolic	Neurology	Obstetrics/Gynecology	Oncology	Ophthalmology	Orthopedics	Psychiatry	Renal	Respiratory	Urology	Not specified
Count		5	33	24	4	33	9	18	60	19	41	1	95	10	1	17	1	15	4	1
Peak sales ($MM)	Mean	$2,075	$1,441	$924	$74	$1236	$1173	$2025	$1218	$368	$973	$165	$1053	$1013	$548	$1694	$142	$811	$584	$148
	Std	$3780	$1700	$877	$45	$1869	$1900	$2945	$2441	$445	$1375		$1871	$2067		$1773		$731	$293	
Pecentiles	5%	$52	$85	$65	$36	$50	$62	$79	$27	$45	$33	$165	$35	$41	$548	$40	$142	$58	$304	$148
	25%	$169	$258	$420	$53	$191	$140	$178	$107	$136	$230	$165	$150	$65	$548	$462	$142	$189	$369	$148
	50%	$480	$852	$790	$63	$646	$211	$400	$385	$217	$484	$165	$419	$103	$548	$1129	$142	$911	$567	$148
	75%	$892	$1,861	$1115	$84	$1452	$779	$2890	$1030	$417	$1156	$165	$1311	$440	$548	$2117	$142	$1140	$782	$148
	95%	$7227	$4,981	$2489	$127	$4285	$4544	$8701	$5154	$1122	$3858	$165	$4061	$4647	$548	$5331	$142	$2128	$889	$148
Time to peak sales (Years)	Mean	3.8	8.0	8.1	4.5	5.9	5.1	6.6	5.7	5.7	6.0	4.0	6.4	4.7	12.0	7.3	6.0	6.3	7.8	4.0
	Std	1.9	3.8	2.9	2.5	2.9	1.3	3.3	3.6	2.2	2.6		3.3	2.5		3.2		3.2	1.0	
Pecentiles	5%	2.2	4.0	4.2	2.3	3.0	4.0	4.0	2.0	2.9	3.0	4.0	2.0	2.5	12.0	2.8	6.0	2.7	7.0	4.0
	25%	3.0	6.0	5.8	3.5	4.0	4.0	4.0	3.0	4.0	4.0	4.0	4.0	3.3	12.0	5.0	6.0	5.0	7.0	4.0
	50%	3.0	7.0	8.5	4.0	5.0	5.0	6.0	5.0	5.0	5.0	4.0	6.0	4.0	12.0	8.0	6.0	5.0	7.5	4.0
	75%	4.0	10.0	9.0	5.0	8.0	6.0	6.0	7.0	7.0	7.0	4.0	8.0	5.0	12.0	9.0	6.0	7.0	8.3	4.0
	95%	6.4	16.0	13.9	7.4	11.2	7.0	12.6	13.1	9.1	11.0	4.0	12.0	8.7	12.0	12.2	6.0	11.1	8.9	4.0
5-Year CAGR (%)	Mean	19%	41%	54%	1%	52%	51%	55%	29%	34%	50%	18%	35%	21%	20%	45%	13%	48%	53%	− 12%
	Std	36%	28%	43%	23%	46%	20%	45%	82%	53%	47%		92%	20%		49%		56%	15%	
Pecentiles	5%	-13%	1%	8%	− 19%	− 14%	23%	12%	− 58%	− 10%	− 5%	18%	− 29%	− 4%	20%	− 2%	13%	− 27%	35%	− 12%
	25%	-4%	25%	30%	− 14%	21%	37%	20%	− 12%	16%	30%	18%	7%	10%	20%	13%	13%	19%	52%	− 12%
	50%	15%	39%	49%	− 4%	44%	53%	38%	17%	24%	43%	18%	22%	19%	20%	31%	13%	31%	59%	− 12%
	75%	24%	58%	62%	11%	87%	66%	86%	45%	36%	67%	18%	45%	35%	20%	55%	13%	85%	60%	− 12%
	95%	68%	81%	125%	28%	127%	74%	134%	104%	67%	148%	18%	89%	49%	20%	143%	13%	134%	61%	− 12%
Cluster count (%)	Early, sharp peak	0%	3%	0%	25%	0%	0%	0%	22%	0%	2%	0%	8%	10%	0%	6%	0%	7%	0%	0%
	Fast uptake, Early plateau	80%	15%	17%	50%	33%	44%	28%	32%	42%	37%	100%	35%	50%	0%	29%	0%	27%	0%	100%
	Moderate uptake	0%	36%	21%	0%	39%	44%	50%	25%	47%	39%	0%	28%	30%	0%	29%	0%	40%	50%	0%
	Prolonged uptake	20%	45%	63%	25%	27%	11%	22%	22%	11%	22%	0%	28%	10%	100%	35%	0%	27%	50%	0%

### Drug Modality

We stratified drug products in our database by type of therapeutic agent, including categories of traditional small molecules, monoclonal antibodies, peptides, vaccines, cell therapies, gene therapies, oligonucleotides (including antisense and siRNA), steroids, and others (detailed statistical analysis shown in Table 3). We report top-line findings for categories with n > 10 entries:

- *Small molecules* constitute the majority of drug launches (239 out of 391) in our database, and have a median peak sales of ~ $423M; median time-to-peak is ~ 6 years. This indicates that “typical” small-molecule drug tends to have relatively moderate commercial success. Small molecules include products indicated for a diverse range of conditions; some become blockbusters, however, many have relatively modest peaks due to competition. The median 5—yr CAGR of ~ 34% suggests healthy early growth on average, but not as steep as some biologics.
- *Monoclonal antibodies (mAbs)*, which include many biologic therapies, maintain relatively high median peak sales of ~ $973M (n = 61). Biologics are often engineered to target high-unmet-need conditions, thereby commanding premium pricing. Interestingly, the median time to peak for mAbs is ~ 6 years, similar to small molecules. This suggests that while antibodies generally reach higher peaks, mAbs do not necessarily reach them faster than small molecules. The early growth (median ~ 30% CAGR) is robust.
- *Peptides* (e.g., peptide hormones) have a median peak sales of ~ $512M, and a time-to-peak of ~ 5 years (n = 18); this category includes drugs such as insulin analogs, GLP-1 agonists, etc. Peptides often target large patient populations (diabetes, etc.), which is a contributor to the the relatively high peak and fast growth (median ~ 40% 5–yr CAGR; rapid uptake in newer diabetes peptide treatments have been experiencing rapid uptake)
- *Vaccines* (n = 16) have a relatively moderate median peak ~ $556M, but a median time to peak of ~ 7 years and a median 5–yr CAGR of ~ (4)%. The negative median growth rate likely reflects that many vaccine products have an initial bolus of sales (if a vaccine addresses a large susceptible population, launches usually feature a “catch-up” campaign) and then sales level off, growing only with population birth rates. In other words, a vaccine might launch and quickly immunize a large group (yielding high early sales, possibly even peak in the first couple of years), and thereafter sales are steady or decline. The data suggests that for many vaccines, sales by year 5–7 are already near their peak levels (hence little further growth). This dynamic is distinct from therapeutics which often gradually build patient share. Nonetheless, the relatively high median peak of ~ $556M indicates vaccines can be quite lucrative (consider products like HPV vaccines, pneumococcal vaccines, etc.). Notably, if COVID-19 vaccines were in the dataset (some launched 2021), they would exhibit extremely large peaks very quickly; however, our inclusion criteria (5 years of sales) would exclude those recent launches.

**Table 3:** Statistical analysis for drug product modality stratification

Key metric	Statistic	Antisense	Carbohydrate, glycoprotein, or glycopeptide	Cellular	Monoclonal antibody	Natural protein	Oligonucleotide	Peptide	Protein	Small molecule	Steroid	Vaccine	Viral gene therapy	siRNA/RNAi	Not specified
Count		3	3	4	61	1	2	18	35	239	4	16	2	1	2
Peak sales ($MM)	Mean	$570	$425	$375	$1827	$22	$113	$589	$1203	$1059	$491	$951	$292	$268	$1342
	Std	$455	$634	$328	$2576		$41	$719	$2182	$1742	$550	$1203	$298		$609
Pecentiles	5%	$171	$35	$73	$47	$22	$88	$37	$44	$29	$27	$167	$103	$268	$954
	25%	$336	$60	$179	$274	$22	$99	$146	$143	$155	$96	$272	$187	$268	$1126
	50%	$541	$91	$321	$954	$22	$113	$512	$219	$423	$369	$556	$292	$268	$1342
	75%	$790	$624	$517	$2168	$22	$128	$725	$814	$1245	$764	$940	$398	$268	$1557
	95%	$989	$1050	$752	$6753	$22	$139	$1324	$6131	$3974	$1127	$2849	$482	$268	$1729
Time to peak sales (Years)	Mean	4.7	6.7	4.8	7.1	3.0	4.5	5.3	6.8	6.2	8.8	7.2	3.0	5.0	5.0
	Std	2.1	2.1	1.5	3.4		0.7	2.9	4.1	2.9	5.0	4.6	1.4		0.0
Pecentiles	5%	3.1	5.1	3.2	3.0	3.0	4.1	2.9	2.0	2.0	5.2	2.0	2.1	5.0	5.0
	25%	3.5	5.5	3.8	5.0	3.0	4.3	3.0	4.0	4.0	5.8	3.8	2.5	5.0	5.0
	50%	4.0	6.0	5.0	6.0	3.0	4.5	5.0	6.0	6.0	7.0	6.5	3.0	5.0	5.0
	75%	5.5	7.5	6.0	9.0	3.0	4.8	6.8	9.0	8.0	10.0	8.8	3.5	5.0	5.0
	95%	6.7	8.7	6.0	13.0	3.0	5.0	8.9	16.0	12.0	14.8	15.8	3.9	5.0	5.0
5-Year CAGR (%)	Mean	78%	55%	17%	36%	15%	27%	43%	30%	45%	32%	1%	14%	27%	82%
	Std	141%	27%	11%	38%		8%	40%	38%	75%	28%	33%	34%		96%
Pecentiles	5%	-9%	35%	10%	0%	15%	22%	− 18%	− 27%	-29%	7%	− 32%	− 7%	27%	21%
	25%	-3%	40%	11%	17%	15%	24%	15%	9%	14%	9%	− 18%	2%	27%	48%
	50%	4%	45%	12%	29%	15%	27%	44%	23%	34%	31%	− 4%	14%	27%	82%
	75%	122%	65%	18%	50%	15%	30%	66%	45%	59%	54%	10%	26%	27%	116%
	95%	217%	81%	30%	80%	15%	33%	106%	99%	131%	58%	49%	36%	27%	143%
Cluster count (%)	Early, Sharp peak	0%	0%	0%	2%	0%	0%	0%	6%	8%	0%	31%	0%	0%	0%
	Fast uptake, Early plateau	33%	0%	50%	33%	100%	100%	50%	31%	29%	25%	38%	50%	100%	50%
	Moderate uptake	67%	67%	0%	36%	0%	0%	28%	29%	34%	25%	6%	50%	0%	50%
	Prolonged iptake	0%	33%	25%	30%	0%	0%	22%	34%	29%	50%	25%	0%	0%	0%

### Sponsor Type

We stratified drug products in our database by whether a top-20 Pharma company (by market cap size) was involved in product sponsorship, see Table 4 for detailed statistical analysis. Drugs launched by top 20 firms have a median peak sales more than double that of drugs from smaller sponsors ($627 M vs $293 M). This is perhaps expected: large pharma companies often have the resources for expansive marketing, pan-geographic reach, and larger pipelines of high-impact drugs. The distribution also showed that the upper end of peak year sales outcomes are much higher for large pharma Cos: for example, the 95th percentile of peak sales for top 20 Pharma-sponsored drugs is ~ $5.4B, versus ~ $2.8B for those not sponsored by a top 20 Pharma Co. Notably, however, among products that achieved regulatory approval while under the sponsorship of a large Pharma Co, most (> 65%) originated from external sources (primarily biotech companies, inorganically acquired by large Pharma Cos) [24]

**Table 4:** Statistical analysis for drug product top 20 Pharma sponsor stratification

Key metric	Statistic	No Top20 pharma sponsor	Top20 pharma sponsor
Count		152	239
Peak sales ($MM)	Mean	$691	$1413
	Std	$1123	$2175
Pecentiles	5%	$24	$42
	25%	$108	$214
	50%	$293	$627
	75%	$783	$1669
	95%	$2792	$5350
Time to peak sales (Years)	Mean	6.4	6.3
	Std	3.4	3.1
Pecentiles	5%	2.6	2.0
	25%	4.0	4.0
	50%	6.0	6.0
	75%	8.0	8.0
	95%	13.5	12.0
5-Year CAGR (%)	Mean	47%	35%
	Std	89%	42%
Pecentiles	5%	− 18%	− 29%
	25%	10%	13%
	50%	29%	32%
	75%	56%	53%
	95%	139%	117%
Cluster count (%)	Early, Sharp peak	5%	8%
	Fast uptake, Early plateau	36%	30%
	Moderate uptake	32%	33%
	Prolonged uptake	26%	30%

When comparing the median time-to-peak for products sponsored by top 20 Pharma vs otherwise, we found that the value was roughly the same for both groups, ~ 6 years. This suggests that large companies do not necessarily achieve peak any faster, on average, despite their marketing muscle. Both large and small players take about 6 years for a product to mature and reach peak sales. Sales growth rates are also similar between both groups, (median ~ 30% 5–yr CAGR). This implies that while top 20 pharma Cos can maximize ultimate sales (through broader reach, more indication expansions, etc.), the basic adoption curve timeline is not dramatically different from those of smaller companies. It’s possible that factors like the therapeutic area or novelty of the drug dictate uptake speed more than the sponsor’s company size.

### Achievement of Blockbuster Status

To directly illustrate the contrast between “blockbusters” and “non-blockbusters”, we stratified the cohort by peak sales level into two groups: those with peak sales < $1 billion (the majority, 273 drugs, ~ 70% of the sample) and those with peak sales ≥ $1 billion (118 drugs, ~ 30%), see Table 5 for detailed statistical analysis.

**Table 5:** Statistical analysis for drug product blockbuster stratification

Key metric	Statistic	PYS < $1000 M	PYS > = $1000 M
Count		273	118
Peak sales ($MM)	Mean	$330	$2988
	Std	$277	$2551
Pecentiles	5%	$26	$1069
	25%	$108	$1363
	50%	$225	$2018
	75%	$503	$3154
	95%	$906	$8861
Time to peak sales (Years)	Mean	5.8	7.6
	Std	3.0	3.4
Pecentiles	5%	2.0	2.0
	25%	4.0	5.3
	50%	5.0	7.0
	75%	7.0	9.0
	95%	11.4	13.0
5-Year CAGR (%)	Mean	38%	46%
	Std	71%	46%
Pecentiles	5%	− 26%	− 19%
	25%	8%	24%
	50%	26%	42%
	75%	51%	64%
	95%	121%	130%
Cluster count (%)	Early, Sharp peak	7%	7%
	Fast uptake, Early plateau	38%	18%
	Moderate uptake	34%	30%
	Prolonged uptake	21%	46%

Not surprisingly, the “blockbuster” cohort had a relatively very high median peak (~ $2B). What’s more interesting is that drugs that eventually become blockbusters, tend to take longer periods of time to reach peak. The median time to peak for ≥ $1B drugs was ~ 7 years, compared to ~ 5 years for sub-$1B drugs. ~ 46% of the blockbuster drugs followed a " slow and steady climb” pattern, whereas only ~ 21% of the non-blockbusters did. Conversely, "delayed acceleration” patterns were far more common among the sub-$1B drugs: ~ 38% of non-blockbusters vs ~ 18% of blockbusters. This makes intuitive sense—a drug with a very large addressable market, likely existing competition, might ramp up more gradually as it penetrates that market or expands indications, whereas a drug with a small niche might reach its full market potential quickly (but that potential is limited in size).

We also see that blockbuster drugs experienced higher early growth (median 42% 5–yr CAGR vs 26% for non-blockbusters). Essentially, blockbusters eventually not only achieved more, but were growing faster in formative years post-launch. The > $1B group also had a wider spread in time to peak (their 75th percentile ~ 9 years vs 7 years for non-blockbusters, with some blockbusters taking up to 13 years to reach peak). In contrast, very few non-blockbusters took beyond 10 years to peak.

These findings suggest a cautionary point, a product that doesn’t feature explosive sales growth in its first few years post-launch, is not necessarily a failure. Conversely, an early fast uptake is not a guarantee of high peak year sales.

### Regulatory Designations

Finally, we stratified our data by whether a drug product received specialty designation (Orphan, Fast Track or Breakthrough) during its regulatory approval/review cycle. These programs are intended to expedite development and regulatory review for drugs addressing unmet medical needs or rare diseases. To note, our observational analysis of expedited regulatory pathways is not intended to draw conclusive causal relationships, but moreso to inform perspectives on the long-term commercial performance of products which receive such regulatory designations. Thus, we analyzed each designation separately, comparing drugs with and without the designation:

- *Orphan*, in our dataset, 124 drugs (33%) had Orphan Drug status. Orphan-designated products indeed have significantly lower median peak sales than non-orphans ($356 M vs $484 M) as shown in Table 6. This is logical since orphan drugs target rare diseases with inherently limited patient populations. Despite often commanding high prices, total sales are often capped by small disease prevalence. Interestingly, the median time to peak is roughly the same (~ 6 years for both groups), indicating that orphan drugs, though serving smaller markets, do not necessarily ramp up faster. Reaching the full patient pool can still take time due to diagnostics, patient finding, and expanding into global markets. Sales growth rates are similar (~ 30% 5-yr CAGR median) for orphan vs non-orphan designated products. However, in terms of uptake patterns, orphan drugs more frequently fell into “slow and steady climb” (32% of orphan vs 27% of non-orphan) and less frequently into “delayed acceleration” (29% vs 34%) clusters. This suggests that even though the target population for orphan drugs are small, these drugs still require time to penetrate as patients are gradually identified or started on therapy. Some orphan drugs may also expand indications or age groups over time, extending their growth phase. In summary, within our analysis, products with orphan designation had smaller commercial peaks but not necessarily shorter timeframes; these products often steadily grow into their niche markets.

**Table 6:** Statistical analysis for drug product top 20 Pharma sponsor stratification

Key metric	Statistic	No orphan	Orphan
Count		267	124
Peak sales ($MM)	Mean	$1203	$980
	Std	$1,835	$1942
Pecentiles	5%	$30	$34
	25%	$169	$144
	50%	$484	$356
	75%	$1406	$1006
	95%	$5004	$2810
Time to peak sales (Years)	Mean	6.2	6.6
	Std	3.2	3.1
Pecentiles	5%	2.0	2.2
	25%	4.0	4.0
	50%	6.0	6.0
	75%	8.0	8.0
	95%	13.0	13.0
5-Year CAGR (%)	Mean	40%	41%
	Std	54%	83%
Pecentiles	5%	− 25%	− 14%
	25%	10%	14%
	50%	31%	33%
	75%	59%	48%
	95%	130%	104%
Cluster count (%)	Early, Sharp peak	7%	6%
	Fast uptake, Early plateau	34%	29%
	Moderate uptake	32%	33%
	Prolonged uptake	27%	32%

- *Fast Track,* 105 drugs (27%) had FDA Fast Track designation (intended to speed development for serious conditions with unmet need) in our dataset, see Table 7 for detailed statistical analysis. Surprisingly, the median peak sales of Fast Track drugs was slightly lower than others ($426M vs $487M; see Table 7). This could be due to the fact just over half (56 of 105; 53.3%) Fast Track therapies are indicated for serious diseases that might are rare. The time to peak, however, was similar for Fast Track vs non-Fast Track drugs (median ~ 6 years). There was no meaningful difference in the sales growth rates (5—yr CAGR) of Fast Track designated drugs. Overall, within our dataset, products with Fast Track designation do not appear to have commercial trajectories (as judged by magnitude, or time to achieving, PYS) that are distinctly different to those of non-Fast Track designated peers.

**Table 7:** Statistical analysis for drug product fast track designation stratification

Key metric	Statistic	No fast track	Fast track
Count		286	105
Peak sales ($MM)	Mean	$1161	$1054
	Std	$1915	$1749
Pecentiles	5%	$34	$22
	25%	$160	$160
	50%	$487	$426
	75%	$1340	$1039
	95%	$4677	$4871
Time to peak sales (Years)	Mean	6.4	6.3
	Std	3.1	3.4
Pecentiles	5%	2.0	2.0
	25%	4.0	4.0
	50%	6.0	6.0
	75%	8.0	8.0
	95%	13.0	12.8
5-Year CAGR (%)	Mean	42%	35%
	Std	64%	65%
Pecentiles	5%	-24%	− 24%
	25%	14%	5%
	50%	33%	27%
	75%	59%	45%
	95%	124%	124%
Cluster count (%)	Early, Sharp peak	6%	10%
	Fast uptake, Early plateau	31%	36%
	Moderate uptake	34%	29%
	Prolonged uptake	29%	26%

- *Breakthrough Therapy*, 68 drugs (18%) in the sample received Breakthrough Therapy (BT) designation, indicating preliminary clinical evidence of a substantial improvement over existing therapies, see Table 8 for detailed statistical analysis. Breakthrough-designated drugs showed a higher median peak ($544M) than non-BT ($426M), and a shorter median time to peak (~ 5 years vs ~ 6 years). This suggests that BT drugs launch with strong demand, featuring heightened sales volume and ramp to peak. Only ~ 18% of BT drugs followed a slow uptake pattern, compared to ~ 31% of non-BT, and a larger share had moderate or fast uptake patterns. About 9% of BT drugs had an early peak (likely those that quickly reached maximums due to finite populations), higher than the 7% for non-BT. The fact that BT drugs achieve higher peaks may reflect that many BT designations have been given to game-changing therapies (e.g., curative treatments, first-in-class oncology agents) that went on to become blockbusters. Their accelerated approval and strong clinical profiles drive rapid uptake. Indeed, by the 75th percentile, BT drugs peaked in ~ 6 years versus ~ 8 years for others. These results indicate that Breakthrough-designated products, compared to those without this designation, end up achieving heightened commercial success (when judged by magnitude and time-to-achieve PYS): such drugs not only address unmet needs but often dominate their markets quickly. The designation itself does not guarantee commercial success, but it is a flag that the drug has exceptional efficacy, which is a likely contributor to high demand.

**Table 8:** Statistical analysis for drug product breakthrough designation stratification

Key metric	Statistic	No breakthrough	Breakthrough
Count		323	68
Peak sales ($MM)	Mean	$1016	$1686
	Std	$1552	$2901
Pecentiles	5%	$29	$41
	25%	$158	$192
	50%	$426	$544
	75%	$1218	$1843
	95%	$4138	$7576
Time to peak sales (Years)	Mean	6.5	5.5
	Std	3.3	2.6
Pecentiles	5%	2.0	2.0
	25%	4.0	4.0
	50%	6.0	5.0
	75%	8.0	6.0
	95%	13.0	9.7
5-Year CAGR (%)	Mean	40%	42%
	Std	51%	108%
Pecentiles	5%	− 20%	− 48%
	25%	11%	12%
	50%	31%	28%
	75%	57%	47%
	95%	124%	124%
Cluster count (%)	Early, Sharp peak	7%	9%
	Fast uptake, Early plateau	32%	35%
	Moderate uptake	31%	38%
	Prolonged uptake	31%	18%

## Discussion

This study provides one of the most comprehensive quantitative assessments to date of pharmaceutical sales trajectories across a large and diverse set of product launches. We found that the distribution of peak sales is extremely broad, spanning from just a few million dollars per year, to well into the tens of billions, with a median of ~ $500 M. Importantly, we showed that a drug’s journey to its peak often takes about ~ 5–6 years on average, but with substantial variance. Roughly half of drugs hit peak sales within ~ 5 years, yet a significant subset require a decade or more to reach full potential. This result challenges the one-size-fits-all notion of the product life cycle; instead, there are multiple successful trajectory shapes.

Through clustering analysis, we identified four clear archetypes of sales uptake: fast vs slow uptake, early peak vs steady plateau. This not only elucidates the heterogeneity in launch patterns, but also provides concrete examples for companies and analysts to use as analogues. Recognizing which archetype a new product is likely to follow can greatly improve forecasting accuracy and strategic planning.

Our stratified findings yield practical insights for strategic decisions. For example, we saw that drugs in therapeutic areas like immunology or respiratory diseases typically achieve higher sales than those in areas like neurology or infectious disease. This underscores why companies often prioritize chronic and large-population diseases for investment – the upside is simply greater. However, we also saw oncology, an area of intense R&D focus, have relatively lower median peak sales per launch, reflecting a highly segmented market. This implies that while oncology will continue to be a major focus (due to medical need and scientific opportunity), from a commercial standpoint a company should like not assume every oncology asset will be a blockbuster. In contrast, the resurgence of metabolic diseases (e.g., obesity treatments) might yield the next wave of blockbusters, as suggested by the high growth rates observed for that category.

One striking insight was the role of sponsoring company size/experience. Despite the resources of large Pharma companies (e.g., vast sales forces, well-developed market expertise, etc.), time to achieve PYS was no shorter than that achieved by smaller companies. This suggests there are limits to how much the adoption curve of a drug product can be compressed; even with ample supporting resources, factors such as clinical data strength, safety profile, and physician/payer conservatism set intrinsic limits to the rates of drug adoption. We found numerous examples wherein smaller companies demonstrated that they can drive rapid uptake, indicating that agility and focus can prove to be a contender against larger-sized competitors. These findings can be used to inform business development and partnership strategies: a small biotech with a novel therapy might not need a large Pharma partner at launch – smaller Cos can achieve near-term penetration on their own or with a small team, especially in concentrated specialty markets.

Our analysis of blockbusters vs non-blockbusters carries important forecasting and investment implications. We quantitatively confirmed a pattern often discussed anecdotally, drugs that become blockbusters usually take longer to get there. Oftentimes, if a new drug sales fail to meet expectations in the first couple of years post-launch, there is pressure to deem those products a disappointment. Our data cautions against prematurely discontinuing a product that shows a “slow uptake” but steady growth – these slow growers may end up as the biggest winners (e.g., many biologics for autoimmune diseases required building treatment paradigms over time but eventually reached multi-billion sales). Conversely, seeing a rapid early uptake should be tempered with the question: is the total market size limited? The fast uptake pattern was much more associated with sub-$1B peak products in our dataset.

The effect of regulatory designations on commercial outcomes are an area of interest for both industry and policymakers. Our findings provide an empirical overview – Fast Track and Breakthrough designations, which signal a drug’s importance and often facilitate earlier approval and tend to yield differentiated sales dynamics. Breakthrough Therapy designation, in particular, appears to be a hallmark of not just clinical promise but also market influence. These drugs significantly outperformed others in peak sales, doing so on a faster timetable. This could be because truly groundbreaking drugs face pent-up demand – once available, they are rapidly adopted. Fast Track status showed quicker time to peak, but ultimately, not higher peak sales; this might suggest that while development was expedited, corresponding market sizes can be reserved in size (many Fast Track drugs address areas like rare cancers or serious infections where uptake is swift among a limited group of patients).

In quantifying the impacts of expedited regulatory pathways on commercial performance, we did not include RMAT (Regenerative Medicine Advanced Therapy) designation. Stratification analysis for RMAT was not captured due to the likely underpowered nature of the data set (recency of RMAT establishment coupled with our 5-year-sales inclusion criteria, leads to a limited set of products for analysis) [25]. We note that research regarding the real-world implications of RMAT designation have been an area of active research. For example, the impacts of RMAT (and other expedited designations) on therapeutic development timelines and sponsor share price have been recently described [26, 27]

### Limitations

While our study is broad, it has important limitations. Our dataset deliberately excluded products with < 5 years of sales data, which means very recent launches (2019 onwards), and any products that failed (removed from market, or sales data not available) quickly post-launch, are not represented. This potentially introduces survivorship bias, however, our work primarily focused on characterizing sales of drugs with known established sales. Sales data integration from EvaluatePharma and Biomedtracker, while comprehensive, may contain reporting inconsistencies or data gaps. We attempted to mitigate this by inflation-adjusting all figures and cross-verifying major outliers, however, this does not entirely eliminate potential errors related to secondary reporting of source data. Similarly, our assignment of primary indication group or modality, do not fully capture the complex dynamics multi-indication drugs or combination therapies perfectly. For example, if a drug launched in one disease and later expanded to another, we still counted it within its first indication’s category for stratification. Another limitation is that we did not explicitly account for competition in each drug’s market. The presence of multiple drugs launching around the same time in a therapeutic area can impact uptake. Our archetypes and medians average over such context-specific scenarios. In practice, forecasting must consider anticipated competition (including generics) within a given indication.

We also did not differentiate by geography – sales were US totals. Different markets (US, EU, etc.) have different adoption curves due to access and reimbursement. A drug might peak later globally than in the US, if European uptake lags; our data, analyzed as aggregates, does not study the dynamics of regional geographies differences.

### Future Directions

In future iterations of our work, we aspire to understand the impacts of multiple other criteria on commercial performance:

- Utilization of novel commercial strategies
- Emerging modalities (e.g., cell and gene therapy) trajectory forecasting, including the influence of RMAT regulatory designations
- Longitudinal analyses
- Sensitivity to launch year (older launches vs recent trends)
- Correlation between number of patients in trials vs. future sales trajectories
- Further sponsor stratification: combined sponsorship between top20 PharmaCo and biotech
- Commercial sales within geographies outside the US

Additionally, we aim to employ multivariable regression statistical analysis to further isolate the impacts of individual product-specific characteristics on commercial performance.

## Conclusion

In summary, this study provides a robust, data-driven characterization of pharmaceutical sales trajectories, offering valuable benchmarks and archetypes. We demonstrated that sales uptake curves are far from uniform – they range from rapid ascents to prolonged climbs – and that factors such as therapeutic area, modality, and regulatory status are key determinants of such patterns. By quantifying metrics like time-to-peak and peak sales across hundreds of drug launches, we deliver practical reference points for forecasting and strategy.

Our findings have direct implications for pharmaceutical forecasting and decision-making. Companies and investors can calibrate expectations for new product launches against historical realities, improving planning accuracy. The identification of distinct uptake archetypes equips stakeholders to anticipate how a product might behave in the market, and strategize accordingly (for example, allocating resources for a long market-building phase versus capitalizing quickly on a fleeting opportunity). Furthermore, this work highlights the importance of patience and long-term investment for therapies that address large populations or novel mechanisms – some of the most impactful drugs needed many years to realize their full market potential. Additionally, our work underscores the necessity of swift and efficient commercialization for drugs aimed at smaller or fast-saturating markets.

Ultimately, by illuminating the empirical patterns of how drug sales evolve, this study contributes to a more evidence-based approach in an area that has often relied on intuition and over-optimistic projections. These insights enhance our collective ability to bring new therapies to patients successfully by aligning scientific innovation with realistic commercial execution and expectations. We envision that the quantitative benchmarks established here will become a reference toolkit for the industry, aiding in everything from internal budget planning to external stakeholder communications. In a field where the stakes of getting it right or wrong are measured in billions of dollars and, more importantly, in patient health outcomes, such clarity is invaluable. Our analysis reinforces that while every new drug is unique, few are completely unprecedented – their trajectories tend to follow patterns we can learn from. Leveraging those lessons will be crucial for the next generation of biomedical innovation and the sustainable success of the biopharmaceutical enterprise.

## Data Availability

Our analysis primarily sources historic company financial data from Biomedtracker (BMT, Norstella Companies, Yardley, PA). We also obtain essential drug metadata – including indication, modality, approval year, sponsor, and regulatory pathway – from BMT’s comprehensive pharmaceutical database, which provides standardized and aggregated drug development information. For instances of data missingness, we improve the completeness of our database by integrating Evaluate Pharma (Norstella Companies, Yardley, PA). Drug product matching across data sets is completed using exact matches on brand name and supplemented with manual confirmations of highly similar brand name matches as determined by algorithmic approximate string matching. To note, both Biomedtracker and Evaluate Pharma utilize primary sources including, but not limited to, company presentations, SEC filings (10-K and 10-Q reports), annual reports, and other public financial disclosures to generate a repository of geography-specific historic sales data. We adjusted all sales figures in our database to be inflation-adjusted on an annual basis utilizing the NIH Biomedical Research and Development Price Index.

## Footnotes

1 Year 1, the year of approval is not included as it is often a stub year.

2 For reference, iff we expand the window of consideration to only include drug products approved in 2015 or earlier (implying > 10 years of sales data), the following statistics are observed: [n = 251 products], [mean = $1339 M], [STD = $1997 M], [min = $10 M], [5th percentile = $48 M], [25th percentile = $264 M], [median = $627 M], [75th percentile = $1485 M], [95.^th^ percentile = $5,160 M], [max = $15,114 M].
